# The smallest tetrapod from the Middle Triassic of South America: a new procolophonoid parareptile from the Ladinian of Southern Brazil

**DOI:** 10.1038/s41598-026-35114-3

**Published:** 2026-01-28

**Authors:** Rodrigo T. Müller, Lúcio Roberto-da-Silva, Pedro Lucas Porcela Aurélio, Leonardo Kerber

**Affiliations:** 1 Centro de Apoio À Pesquisa Paleontológica da Quarta Colônia, Universidade Federal de Santa Maria, Rua Maximiliano Vizzotto, 598, São João do Polêsine, Rio Grande do Sul 97230-000 Brazil; 2 Programa de Pós-Graduação em Biodiversidade Animal, Universidade Federal de Santa Maria, Santa Maria, Rio Grande do Sul 97105-120 Brazil; 3 Escola Estadual de Ensino Médio Virgilino Jayme Zinn, 24ª Coordenadoria Regional de Educação, Cachoeira do Sul, Rio Grande do Sul 96505-110 Brazil; 4 Cachoeira do Sul, Brazil

**Keywords:** Ecology, Ecology, Evolution, Zoology

## Abstract

The Middle Triassic fossil record of South American parareptiles is scarce, with only a few procolophonoid specimens known. Here, we describe *Sauropia macrorhinus* gen. et sp. nov., a procolophonoid from the Ladinian (Pinheiros-Chiniquá Sequence of the Santa Maria Supersequence) of southern Brazil. The holotype, a nearly complete skull measuring only 9.5 mm in length, represents the smallest tetrapod known from these deposits. Its unique combination of features includes a proportionally large external naris, slender dorsal ramus of the maxilla, broad interorbital space, and three premaxillary teeth. Phylogenetic analyses consistently recover *Sauropia macrorhinus* gen. et sp. nov. as an early-diverging procolophonid, although its phylogenetic placement is complicated by its putatively immature ontogenetic stage. Its morphology provides valuable insight into early developmental stages of parareptiles and contributes to the understanding of Middle Triassic terrestrial ecosystems. Based on size and dentition, the new taxon was likely insectivorous or fed on other small invertebrates, possibly being predated upon by small carnivorous organisms. This discovery expands the taxonomic and ecological diversity known for Middle Triassic faunas of South America and enhances our understanding of the structure and complexity of terrestrial food webs in Middle Triassic ecosystems, preceding the Carnian Pluvial Episode and the rise of dinosaurs.

**Supplementary Information:**

The online version contains supplementary material available at 10.1038/s41598-026-35114-3.

## Introduction

The Triassic Period marks the recovery from the most drastic mass extinction that ever struck the history of life on Earth^1^. Biodiversity flourished, giving rise to some of the most iconic groups of vertebrates, including dinosaurs, pterosaurs, and a variety of bizarre groups^2–6^. Whereas the Triassic witnessed the emergence of several new groups, it also marked the end of some groups that had managed to survive the Permian–Triassic extinction event, including the parareptiles^7^. Procolophonoids were the last surviving members of Parareptilia, represented during the Triassic Period by owenettids^8–11^ and procolophonids^12–14^. Owenettidae was a relatively low-diversity clade^15^, with its youngest representatives going extinct in the early Late Triassic^9^. Conversely, Procolophonidae was far more diverse, both taxonomically and morphologically^16^. Throughout their evolutionary history, procolophonids dispersed across Pangaea and developed a variety of feeding strategies^17^. Nevertheless, the clade became extinct before the end of the Triassic Period, persisting until the late Norian^13^.

Procolophonids are abundant in the fossil record of Early Triassic beds in Brazil^17–21^. On the other hand, the fossil record from Brazilian Middle Triassic deposits is much scarcer, currently represented by a single specimen^22^. By the Late Triassic, procolophonids become relatively more common than they were in the Middle Triassic, though they remain less abundant and diverse than in the Early Triassic record^12,23,24^. With regard to Owenettidae, the clade is recorded in Brazil solely from Middle Triassic deposits^8,9,15^, and is completely absent from both Early and Late Triassic strata. In the present study, we describe a new procolophonoid from Middle Triassic deposits of Brazil. The unusual osteology of this new parareptile is described, and its phylogenetic affinities are investigated. The specimen is particularly interesting for representing the smallest tetrapod ever discovered from these deposits.

## Material and methods

### Institutional abbreviations

BRSUG, University of Bristol, Geological Collection, Bristol, UK; CAMZM, Museum of Zoology, Cambridge University, Cambridge, UK; CAPPA/UFSM, Centro de Apoio à Pesquisa Paleontológica da Quarta Colônia/Universidade Federal de Santa Maria, São João do Polêsine, Brazil; FC-DPV, Departamento de Paleontología de Vertebrados, Facultad de Ciencias, Montevideo, Uruguay; NSM, Nova Scotia Museum, Halifax, Canada; UFSM, Laboratório de Estratigrafia e Paleobiologia, Universidade Federal de Santa Maria, Santa Maria, Brazil; YPM, Yale Peabody Museum, New Haven, USA.

### Specimen

The holotype is housed at the collection of the Centro de Apoio à Pesquisa Paleontológica da Quarta Colônia da Universidade Federal de Santa Maria (CAPPA/UFSM), in the municipality of São João do Polêsine, Rio Grande do Sul, Brazil. Its collection number is CAPPA/UFSM 0510 and consists of an almost complete skull with mandible in occlusion.

### CT scanning and 3D model generation

CAPPA/UFSM 0510 was scanned using a Nikon XT H 225 micro-CT scanner at the the UFSM facilities. The scan was performed at 150 kV and 180 μA, generating 1007 slices with a voxel size of 0.009 mm. The dataset was processed in Avizo 3D (Thermo Fisher Scientific, Hillsborough, OR, USA). Due to the preservation of the specimen, precise 3D reconstructions of the internal anatomy were not feasible. Therefore, only the tomographic slices were used for interpretative analyses. In addition, the anatomical description was supplemented with a 3D model of CAPPA/UFSM 0510. The specimen was digitally scanned using the Abound v3.1.0 application, generating a model with fewer than 30 million triangles and 8 K textures. The resulting 3D model was then processed using MeshLab v2023.12.

### Phylogenetic analysis

In order to investigate the phylogenetic affinities of the new parareptile, the taxon was included in an updated version of a procolophonoid data matrix^13^. In addition to CAPPA/UFSM 0510, we also incorporated *Macroleter poezicus*, *Owenetta rubidgei*, *Ruhuhuaria reiszi*, and *Cornualbus primus*. The nycteroleterid *Macroleter poezicus* was included to investigate whether using a different outgroup affects the internal affinities of Procolophonoidea. Scores for *Soturnia caliodon* were also updated based on recently published data^24^. The final data matrix comprises 90 morphological characters and 43 operational taxonomic units (OTUs). Both the data matrix and the character list are available as Supplementary Information.

The most parsimonious trees (MPTs) were recovered using TNT version 1.6^25^. Following previous iterations of this data matrix^13^, 10 characters were treated as ordered (i.e., additive): 8, 12, 14, 18, 25, 26, 31, 32, 39, and 81. The use of weighting against homoplasy has been defended by a growing number of studies^26,27^. Therefore, we followed the protocol of Ezcurra et al.^28^, in which an ideal range of concavity constant values (*k*) for a matrix of this size lies between 3 and 8^27^. *Macroleter poezicus* was used to root the MPTs of the six analyses, each of which included 1000 replications of Wagner trees (with random addition sequences), followed by tree bisection reconnection (TBR) branch swapping, holding 10 trees per replicate. Zero-length branches in the recovered MPTs were collapsed, and topologies retained in overflowed replicates were branch-swapped for MPTs using TBR. Consistency index (CI) and retention index (RI) values were calculated using the script by Spiekman and Klein^29^. As in the protocol of Ezcurra et al.^28^, group support was assessed through symmetric resampling analyses with non-zero weights, using 1000 pseudo-replicates. Each pseudo-replicate included 10 Wagner tree replications followed by TBR branch swapping, and both absolute frequencies and group present/contradicted (GC) values were reported. A global strict consensus tree (GSCT) was constructed from all the trees recovered in the six analyses (*k*-values between 3 and 8).

## Nomenclatural acts

This published work and the nomenclatural acts it contains have been registered in ZooBank, the online registration system for the International Code of Zoological Nomenclature. The ZooBank LSIDs (Life Science Identifiers) can be resolved and the associated information viewed through any standard web browser by appending the LSID to the prefix ‘http://zoobank.org/’. The LSID for this publication is: urn:lsid:zoobank.org:pub:2C5CA800-2049-4E7C-8453-3DB2EDC0AD05.

## Results

Systematic paleontology

Parareptilia Oslo, 1947

Procolophonoidea Romer, 1956

cf. Procolophonidae Seeley, 1888

*Sauropia macrorhinus* gen. et sp. nov.

[urn:lsid:zoobank.org:act:ED9B5A72-8105-4EC3-B7A1-39251D2FDE30 (genus)]

[urn:lsid:zoobank.org:act:8D3CB8D0-B8A9-4DAB-B7C4-444FF5D0BE11 (species)]

*Holotype* CAPPA/UFSM 0510, an almost complete skull with mandible in occlusion.

*Etymology* The genus name combines the Greek word “sauros” (= lizard) and the Portuguese word “piá” (= young boy), a regional term from southern Brazil rooted in Gaúcho culture, particularly in the state of Rio Grande do Sul, where it is commonly used to refer to a child. The name alludes to the small size and putative early ontogenetic stage of the holotype. The specific epithet combines the Greek words “makros” (= large) and “rhinos” (= nose or snout), in reference to the proportionally enlarged external naris of the holotype.

**Fig. 1:**
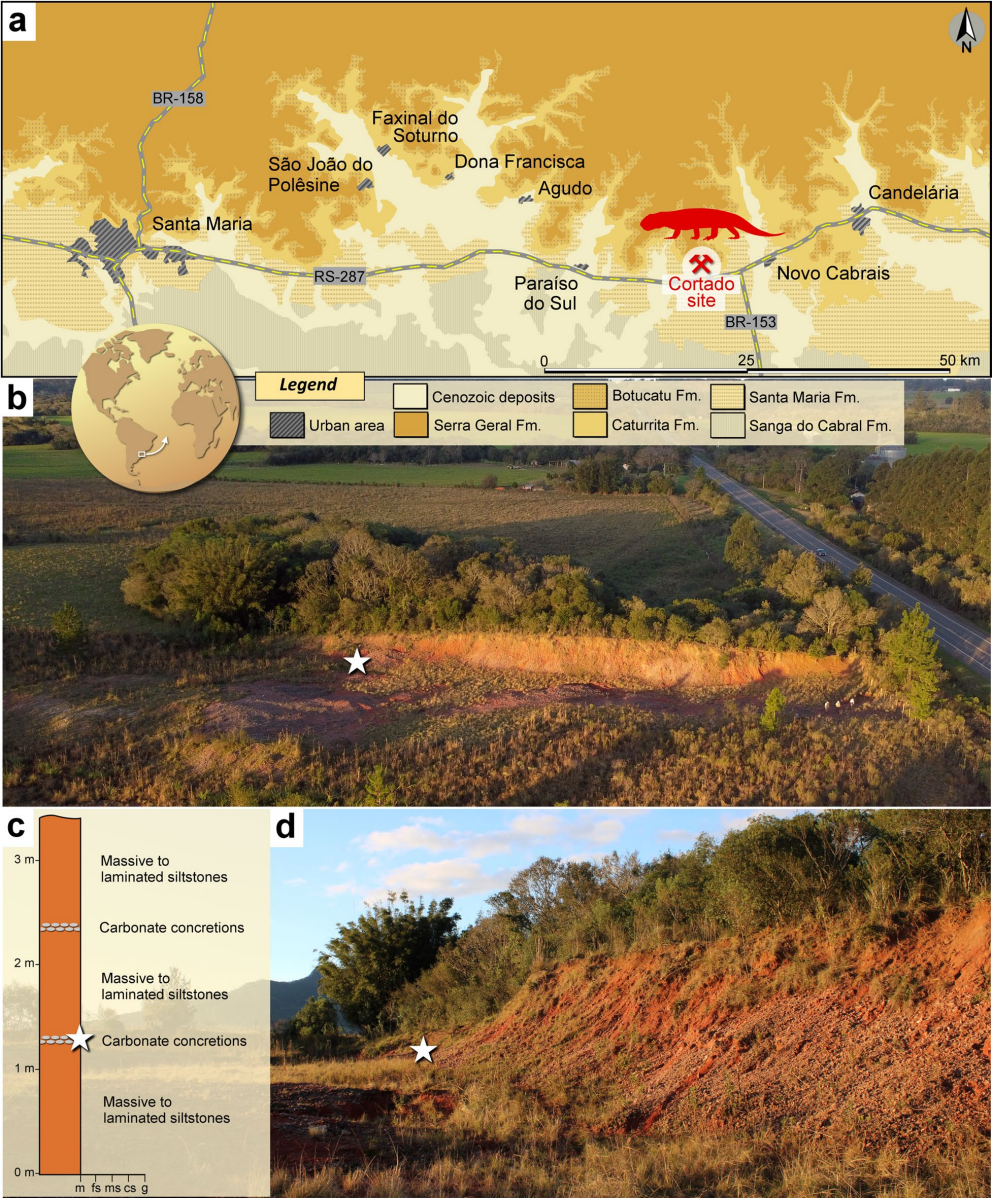
Provenance of *Sauropia macrorhinus* gen. et sp. nov. (**a**) Location and geological context of the Cortado site, Novo Cabrais, Rio Grande do Sul, Brazil. Map was generated with GIMP version 2.8 (https://www.gimp.org/). (**b**) Aerial view of the Cortado site (photograph taken in June 2024). The white star marks the location of CAPPA/UFSM 0510. (**c**) Stratigraphic column of the Cortado site^30^ showing the level where CAPPA/UFSM 0510 (white star) was discovered. (**d**) General view of the Cortado site (taken in June 2024), indicating the spot where CAPPA/UFSM 0510 was discovered (white star).

*Type Locality, age, and horizon* Cortado site (29°44′52″S, 53°01′48″W), municipality of Novo Cabrais, Rio Grande do Sul, Brazil (Fig. 1)^30^. This site belongs to the Pinheiros-Chiniquá Sequence of the Santa Maria Supersequence, Santa Maria Formation, Paraná Basin^31^. The fossiliferous content of the Cortado site belongs to the *Dinodontosaurus* Assemblage Zone (AZ)^30,32^. This AZ is Ladinian (Middle Triassic) in age, based on radioisotopic data^33^.

*Diagnosis* *Sauropia macrorhinus* differs from all other known procolophonoids with comparable material in (*local autapomorphies): Skull nearly as wide as it is long; proportionally large external naris (taller than the orbitotemporal fenestra)*; broad interorbital space in dorsal view; posterior margin of the orbitotemporal fenestra almost reaching the posterior end of the skull; premaxillae bearing three teeth; slender dorsal ramus of the maxilla*; absence of a temporal fenestra bordered anteriorly by the postorbital; U-shaped mandible in ventral view; and anterior maxillary teeth with a circular cross-section (not labiolingually expanded) and lacking a basal constriction.

*Description* The holotype of *Sauropia macrorhinus* preserves most of the skull and mandible. As with most fossils from the *Dinodontosaurus* AZ, parts of the material are covered by concretions and poorly preserve the external bone surface. As a result, several aspects of the craniomandibular anatomy of CAPPA/UFSM 0510 remain obscure, especially bone contacts and finer details. This condition is aggravated by the extremely reduced size of the specimen.

**Fig. 2:**
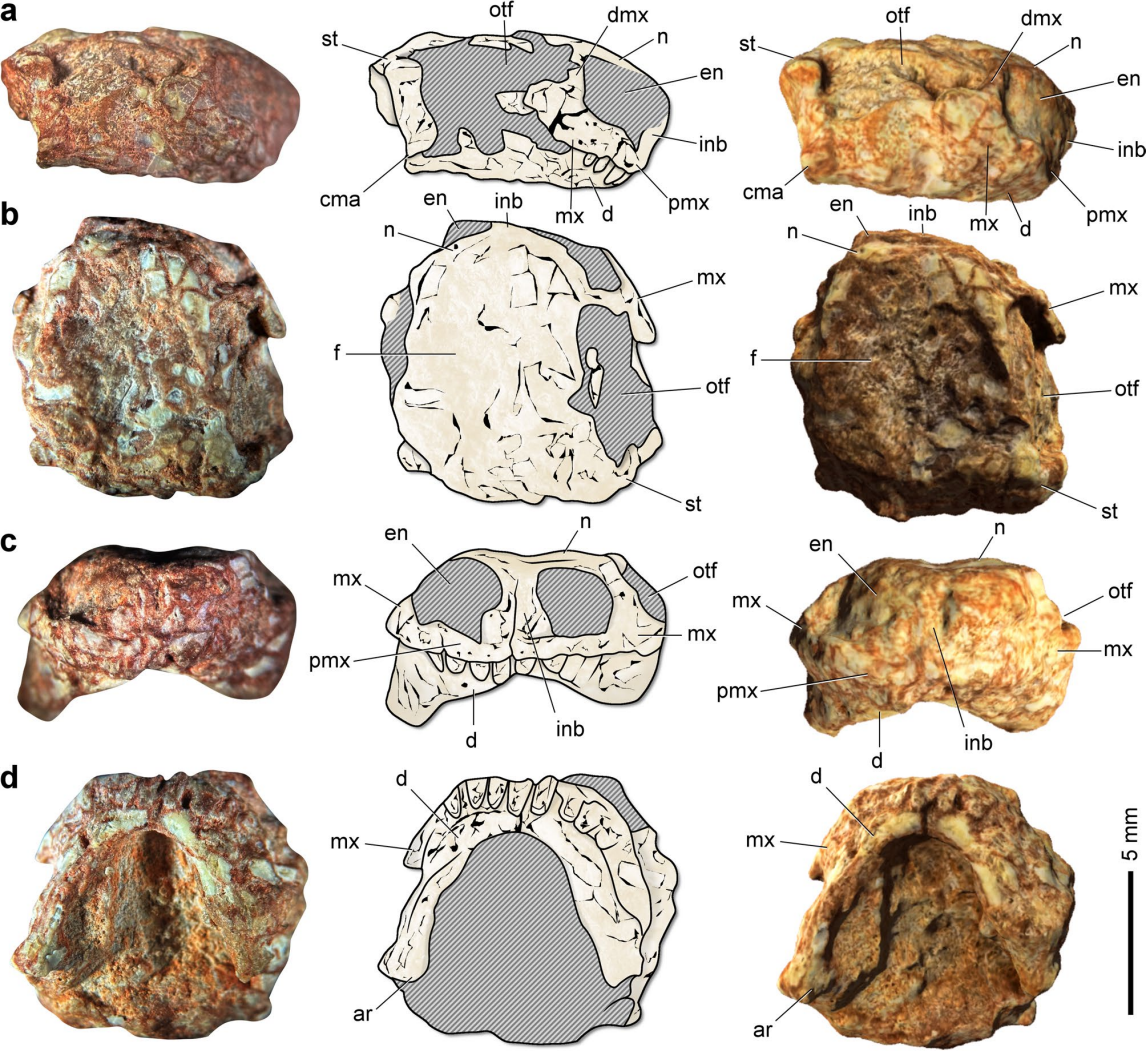
Skull and mandible of *Sauropia macrorhinus* gen. et sp. nov. from the Pinheiros-Chiniquá Sequence (Ladinian) of the Santa Maria Supersequence, southern Brazil. Holotype (CAPPA/UFSM 0510) in right lateral (**a**), dorsal (**b**), anterior (**c**), and ventral (**d**) views. Left panels show photographs, center panels show schematic drawings, and right panels show 3D models. Abbreviations: ar, articular; cma, craniomandibular articulation; d, dentary; dmx, dorsal ramus of the maxilla; en, external naris; f, frontal; inb, internarial bar; mx, maxilla; n, nasal; otf, orbitotemporal fenestra; pmx, premaxilla; st, supratemporal.

The skull of CAPPA/UFSM 0510 is nearly as wide as it is long (Table 1; Fig. 2), which represents an unusual condition among procolophonoids. In dorsal view, the skull is almost rounded, with a broad rostrum (Fig. 2b). The external naris is tall and wide, and the snout is short and deep (height/length ratio = 1.14), a condition more closely resembling that of some procolophonids, such as *Thelerpeton oppressus* and *Phaantosaurus simus*^34^. In contrast, a long and flat snout is present in all owenettids in which this region is preserved^34^. In lateral view, the external naris is elongated (Fig. 2a), a condition shared with owenettids^9,11,15^ and more basal procolophonoids^34^. The orbitotemporal fenestra is also elongated, with its posterior margin nearly reaching the posterior end of the skull. This contrasts with the anteroposteriorly shorter opening observed in owenettids^9,11,15,35^. Moreover, unlike *Candelaria barbouri* (UFSM 11076; UFSM 11131; CAPPA/UFSM 0225)^9,15^, *Sauropia macrorhinus* shows no evidence of a temporal fenestra bordered anteriorly by the postorbital.

**Table 1:** Measurements (in mm) of the skull of *Sauropia macrorhinus* gen. et sp. nov. (CAPPA/UFSM 0510).

Dimension	Measurement
Skull length	9.5
Preorbital skull length	3.4
Maximum diameter of external naris	2.9
Orbitotemporal length	5.7
Maximum temporal width	8.7
Vertical diameter of orbit	1.7
Length of lower jaw	6.2

The main body of the premaxilla is slender and bears three tooth positions (Fig. 2c). This condition resembles that of some procolophonids, such as *Pintosaurus magnidentis* (FC-DPV 1181)^36^, whereas owenettids possess at least five tooth positions^9,11^. The premaxilla is downturned (Fig. 2a), resulting in an oblique alveolar margin. In anterior view, the internarial bar is relatively wide ventrally and tapers dorsally (Fig. 2c). The maxilla is incompletely preserved, lacking its posterior ramus. The anterior ramus is elongated and forms more than half of the ventral margin of the external naris. The dorsal ramus is gracile and constitutes the entire posterior border of the external naris (Fig. 2a). In *Candelaria barbouri* (UFSM 11076; UFSM 11131; CAPPA/UFSM 0225)^9,15^, the dorsal ramus is anteroposteriorly broder, giving it a more robust appearance. There is no evidence of a maxillary depression on the anterior portion of the dorsal ramus. The contact between the maxilla and the lacrimal is poorly preserved.

The extremities and contacts between the bones forming the skull roof are poorly preserved, hindering detailed observations. One of the most distinctive features of this region is the broad area between the orbitotemporal fenestrae (Fig. 2b). This condition suggests proportionally wide frontals, differing from those of other procolophonoids. Nevertheless, the potential ontogenetic influence on this trait should be considered. The bones bordering the dorsal rim of the orbitotemporal fenestrae are dorsally projected, forming a faint longitudinal crest along the lateral edges of the skull. In contrast, the midline of the skull is longitudinally depressed, resulting in a concave region visible in anterior view. The position of the pineal opening is unclear. The posterolateral corner of the supratemporal is rounded (Fig. 2b).

The basicranium and palate are poorly preserved, which prevents access to most anatomical details. However, it is possible to observe that the ventral surface of the parabasisphenoid lies well above the ventral margin of the quadrate. The presence and configuration of palatal teeth remain uncertain.

Both hemimandibles are preserved and remain in occlusion. In ventral view, the mandible is horseshoe-shaped, with the anterior extremities of the dentaries curving medially to contact each other at a short symphysis (Fig. 2d). The broad, U-shaped anterior portion of the mandible in *Sauropia macrorhinus* differs from the proportionally narrower, V-shaped mandibles of some procolophonids in ventral view, such as *Kapes bentoni* (BRSUG 29950-13)^37^ and *Cornualbus primus* (UFSM 11607)^22^. A comparable U-shaped anterior portion of the mandible can also be observed in the owenettid *Ruhuhuaria reiszi* (CAMZM T997)^10^ and the procolophonid *Hypsognathus fenneri* (YPM 55831; NSM 998GF45.1)^38^. The ventral surface of the anterior end of the dentary is straight in lateral view (Fig. 2a), lacking any ventral projection. In *Scoloparia glyphanodon* (YPM VPPU 024501)^39,40^ and *Hypsognathus fenneri* (YPM 55831)^38^, a faint ventral projection is present in this region. Most of the ventral surface of both hemimandibles is also straight, with no ventral convexity along the posterior half. The craniomandibular articulation lies approximately at the same level as the preserved maxillary tooth row (Fig. 2a).

**Fig. 3:**
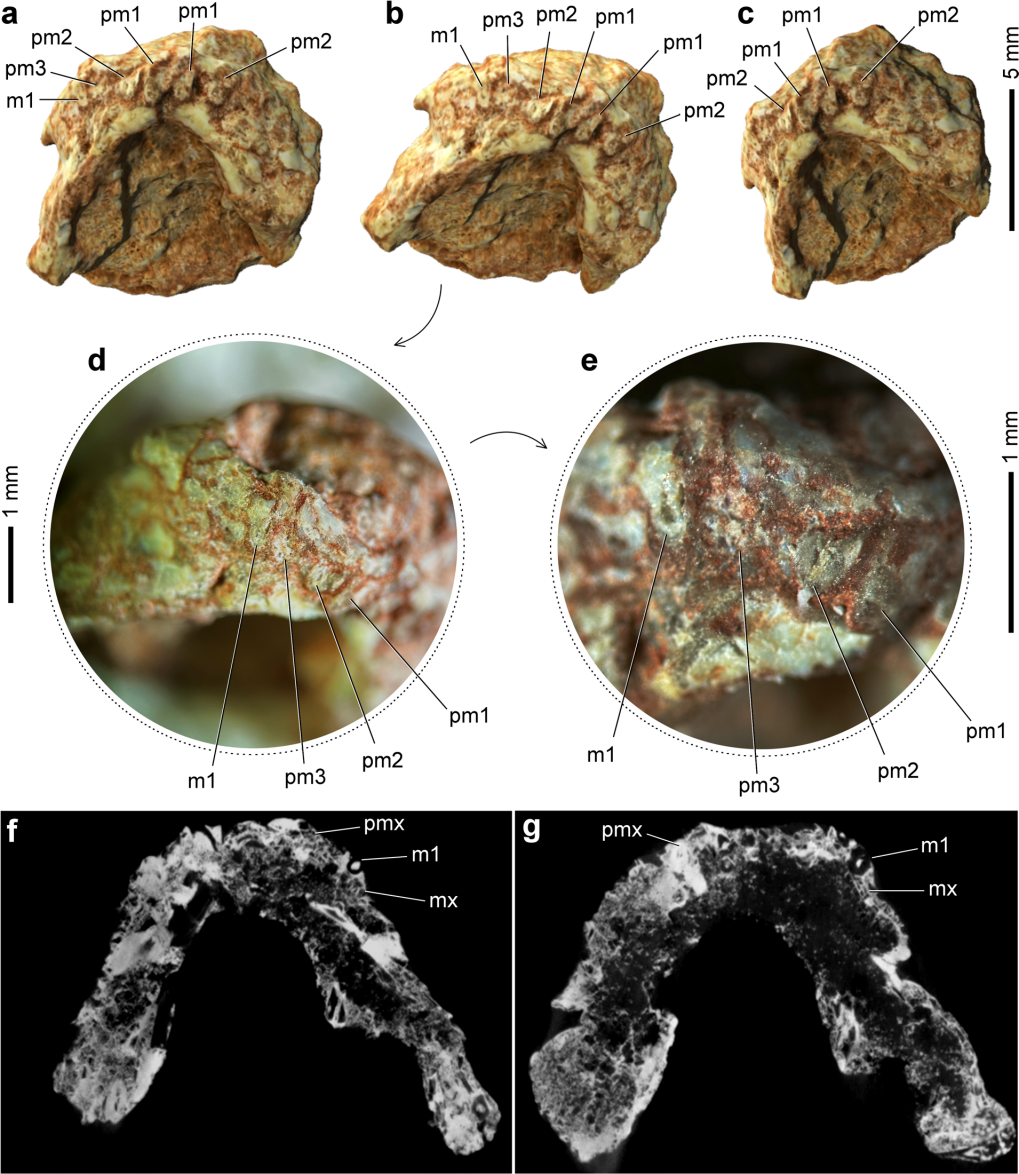
Dentition of *Sauropia macrorhinus* gen. et sp. nov. from the Pinheiros-Chiniquá Sequence (Ladinian) of the Santa Maria Supersequence, southern Brazil. 3D model of the holotype (CAPPA/UFSM 0510) in anteroventral (**a**), right lateroventral (**b**), and left lateroventral (**c**) views. Photograph of the right premaxilla and maxilla of CAPPA/UFSM 0510, magnified, in anterolateral view (**d**). Details of the right premaxillary and maxillary dentition of CAPPA/UFSM 0510 in anterolateral view (**e**). Transverse slices of CAPPA/UFSM 0510 in (**f**) and (**g**). Abbreviations: m, maxillary tooth; mx, maxilla; pm, premaxillary tooth; pmx, premaxilla.

Regarding the dentition, CAPPA/UFSM 0510 preserves three teeth in each premaxilla (Fig. 3a) and only one tooth in the right maxilla (Fig. 3b). Therefore, the total number of maxillary teeth is uncertain. Similarly, the total number and shape of the dentary and palatal teeth remain unknown. The premaxillary teeth are relatively large and exhibit a simple morphology (Fig. 3c, d), being straight, with no cusps or basal constriction (Fig. 3e). In cross-section, the premaxillary teeth are circular, resulting in cylindrical crowns. The partially preserved crown of the maxillary tooth suggests a morphology similar to that of the premaxillary teeth, with a circular cross-section (Fig. 3f, g) and the absence of a basal constriction (Fig. 3e).

**Fig. 4:**
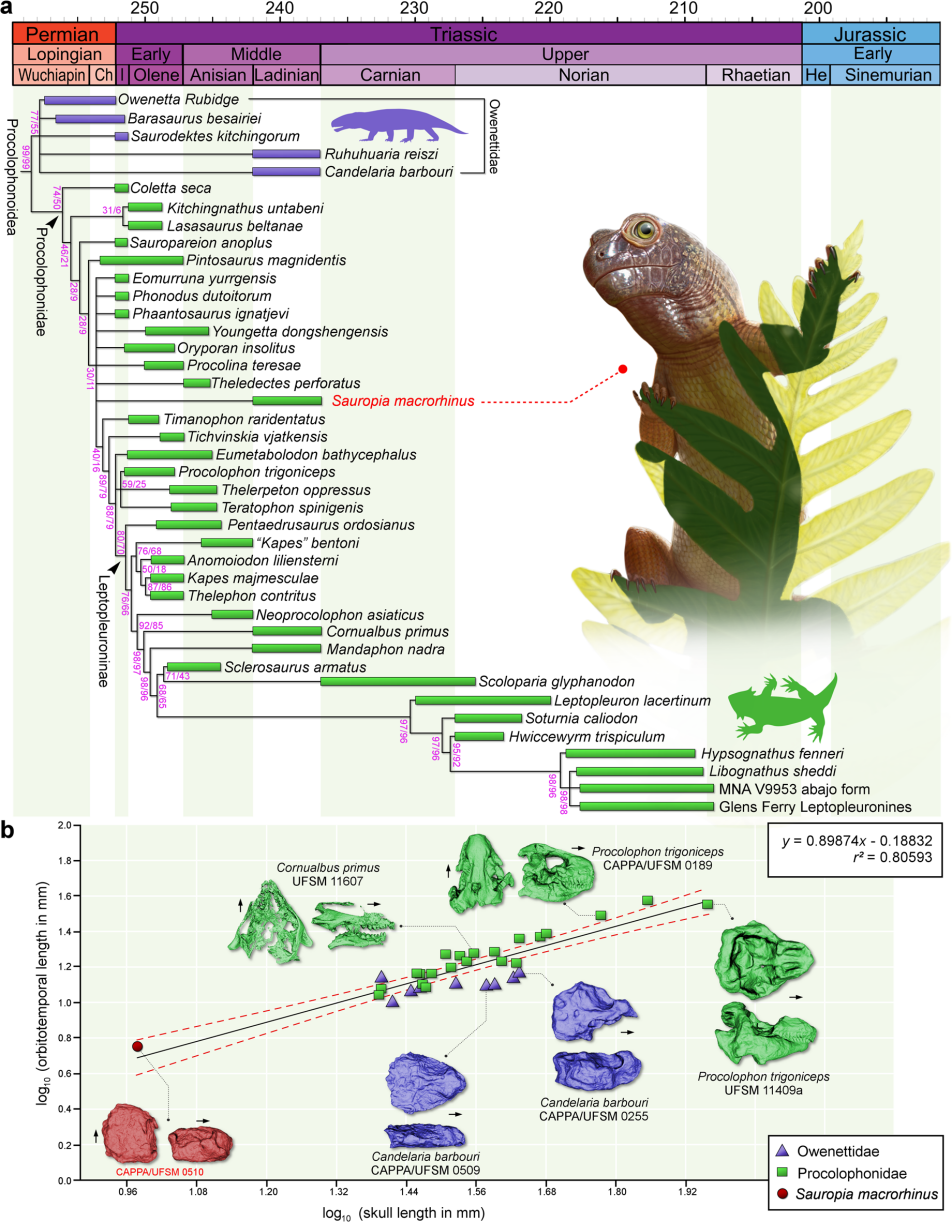
Results of the phylogenetic analysis and ordinary linear regressions. (**a**) Time-calibrated reduced global strict consensus tree depicting the phylogenetic position of *Sauropia macrorhinus* gen. et sp. nov. Values on the branches represent absolute (left) and GC (right) frequencies from symmetric resampling with no-zero weighting. The temporal bars for each OTU represent the maximum and minimum ages of each geological unit. Geological Time Scale was generated with GIMP version 2.8 (https://www.gimp.org/). Artistic representation of *Sauropia macrorhinus* gen. et sp. nov. by Caetano Soares. (**b**) Scatter plots of log-transformed measurements of the skull length and the orbitotemporal length of selected procolophonoids (n = 31). Linear regression line is shown in black, with the 0.95 confidence interval indicated by red dotted lines.

*Phylogenetic analysis* *Sauropia macrorhinus* was recovered as an early-diverging procolophonid in all MPTs from the six analyses (Supplementary Fig. 1–6) using different concavity constant (*k*) values (Table 2). Due to the unstable position of some OTUs, *Sauropia macrorhinus* nests within a large polytomy alongside other unstable procolophonids in the GSCT (Fig. 4a). The inner affinities of owenettids remain unresolved across all analyses, regardless of the *k* value employed. Nevertheless, all OTUs traditionally regarded as owenettids were consistently recovered as such. *Coletta seca*, from the Early Triassic, is recovered as the basalmost procolophonid in all analyses. The clade including *Sauropia macrorhinus* and other procolophonids forms the sister group to *Pintosaurus magnidentis* in the GSCT. Because of its relatively basal position, *Sauropia macrorhinus* is not recovered within any of the less inclusive clades of Procolophonidae (i.e., Theledectinae, Procolophoninae, and Leptopleuroninae).

**Table 2:** Number of most parsimonious trees (MPTs) recovered and homoplasy indices of the six analyses under implied weighting with the different concavity constant values.

Concavity constant value (*k*)	Number of MPTs	Consistence index	Retention index	Fit (adjusted homoplasy)
3	3	0.48936	0.82169	22.97857
4	567	0.49145	0.82344	19.17619
5	567	0.49145	0.82344	16.46032
6	567	0.49145	0.82344	14.42857
7	567	0.49145	0.82344	12.84874
8	567	0.49145	0.82344	11.58384

## Discussion

*Sauropia macrorhinus* exhibits some traits observed in both owenettids and procolophonids. For instance, the maxilla bears a relatively elongated anterior ramus (= premaxillary subnarial process), a condition present in owenettids owenettids^9,11,15^ but absent in most procolophonids^12,13,24,34,36,41^. However, an elongated anterior ramus of the maxilla is not a synapomorphy of Owenettidae, as it also occurs in other procolophonomorphs^42,43^ and is retained in *Colleta seca*^44^, the basalmost procolophonid recovered in the present phylogenetic analysis. Therefore, its presence in owenettids represents a plesiomorphic condition, and its occurrence in *Sauropia macrorhinus* is interpreted as reversal. A similar case concerns the anteroposteriorly elongated external naris. This condition is present in *Sauropia macrorhinus* and owenettids^9,11,15^, whereas in procolophonids the external naris is typically subcircular^34^. Nevertheless, this trait is also plesiomorphic for Owenettidae, as it occurs in earlier procolophonomorphs such as *Nyctiphruretus acudens*^43^ and *Macroleter poezicus*^42^. Thus, the presence of an elongated external naris in *Sauropia macrorhinus* is also interpreted as a reappearance of an ancestral condition. Due to the poorly preserved bone surface, most potential synapomorphies of Owenettidae cannot be properly assessed in CAPPA/UFSM 0510. Conversely, the specimen displays several features found exclusively in procolophonids, including a short and deep snout, an anterodorsally oriented internarial bar (in lateral view), a reduced number of premaxillary teeth, and an elongated orbitotemporal fenestra. The short and deep snout is present in some procolophonids and completely absent in owenettids^34^. However, as snout shape may vary ontogenetically^24,34^, caution is warranted when interpreting this character. Although it appears to be a procolophonid trait among the sampled taxa, its significance is ambiguous due to the putatively immature condition of CAPPA/UFSM 0510. Another feature shared between *Sauropia macrorhinus* and some procolophonids is the anterodorsally oriented internarial bar (= ascending or supranarial process) of the premaxilla. This contrasts with the condition in owenettids^9,11^ and some procolophonids^13,14,36^, where the internarial bar is subvertical at the base and curves dorsally and posteriorly. The presence of three premaxillary teeth in *Sauropia macrorhinus* is likewise shared only with some procolophonids^36,45^. Even four premaxillary teeth are restricted to procolophonids in the current investigated sample, whereas owenettids consistently bear at least five^9,11^. Finally, the elongated orbitotemporal fenestra is another trait shared with some procolophonids. In *Sauropia macrorhinus*, the posterior margin of this fenestra nearly reaches the posterior end of the skull, contrasting with the condition in owenettids, where the fenestra terminates well anteriorly.

Although *Sauropia macrorhinus* was consistently recovered as a member of Procolophonidae in the phylogenetic analysis and shares some unambiguous traits exclusively with members of the clade, its phylogenetic position should be considered tentative due to the putative immature condition of CAPPA/UFSM 0510. It is widely known that ontogeny can produce significant changes in the shape of the vertebrate skeleton^46^. Moreover, the effects of ontogeny on the morphology of procolophonoids remain poorly understood, particularly in owenettids. Some studies have documented ontogenetic changes in snout shape, the number of maxillary teeth, and the morphology of the palate in procolophonids^24,34,47^, whereas well-preserved ontogenetic series remain unknown for owenettids. Therefore, the most unusual features of *Sauropia macrorhinus* should be further investigated as additional specimens become available, allowing for a more detailed examination of the effects of bone maturation on procolophonoid morphology.

There are no unambiguous indicators of the ontogenetic status of CAPPA/UFSM 0510 beyond its extremely small size. Nevertheless, based on comparisons of skull length (9.5 mm) with other procolophonoids, it is highly unlikely that CAPPA/UFSM 0510 represents a skeletally mature individual. For example, the skulls of all examined owenettids and procolophonids are at least 2.5 times longer than that of CAPPA/UFSM 0510, with the most extreme case being a specimen of *Procolophon trigoniceps* (UFSM 11409a)^19^, whose skull is 9.5 times longer. In addition, a one-sample t-test based on the skull lengths of 30 procolophonoid specimens (see Supplementary Information) indicates that CAPPA/UFSM 0510 is statistically smaller than the sample (*p* < 0.0001). It is expected that even the largest procolophonoids developed from relatively small early ontogenetic stages. Therefore, the discovery of a 9.5 mm-long skull of a procolophonoid provides valuable information on the development of this important group of parareptiles. When plotted in a linear regression comparing total skull length and orbitotemporal length (Fig. 4b), the holotype of *Sauropia macrorhinus* falls within the 95% confidence interval above the regression line, consistent with the condition observed in several procolophonids. In contrast, nearly all owenettids plot far from the regression line, falling even below the 95% confidence interval, with the exception of *Ruhuhuaria reiszi*. Thus, even representing an extremely reduced specimen, the orbitotemporal fenestra of CAPPA/UFSM 0510 is not inconsistent with the expected length for procolophonids. On the other hand, if *Sauropia macrorhinus* is interpreted as a skeletally immature owenettid, it would suggest a potential allometric growth pattern, with the fenestra becoming proportionally smaller during development.

**Fig. 5:**
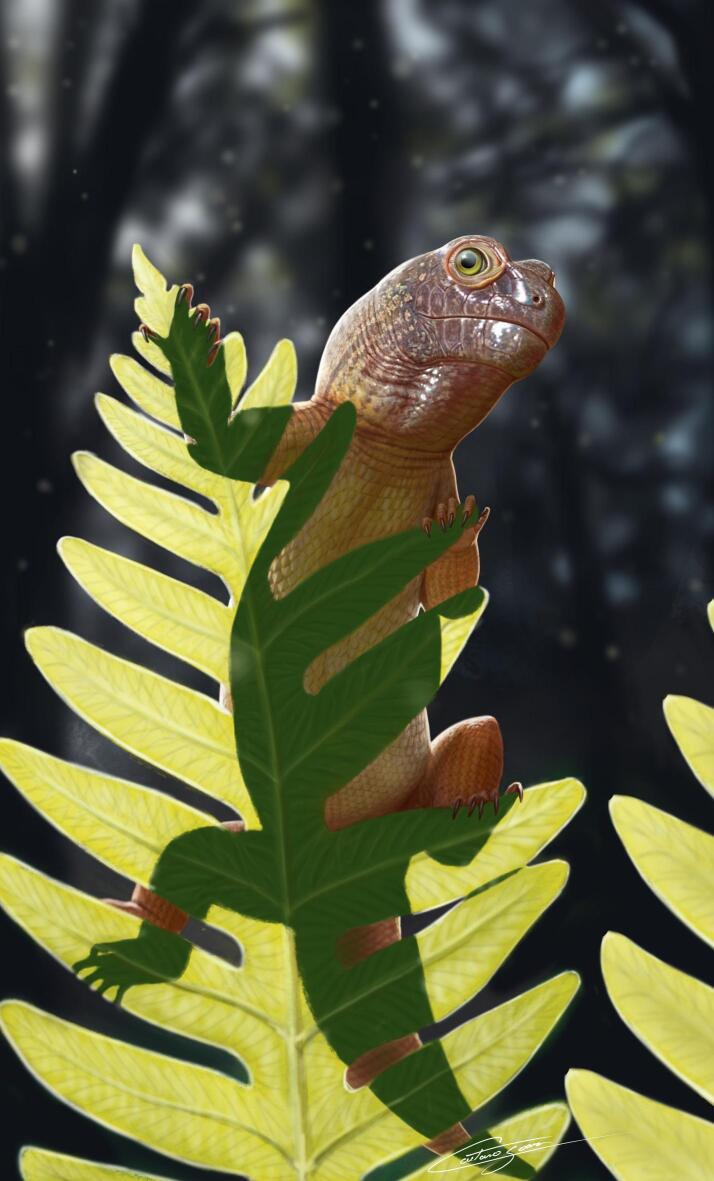
Artistic representation of *Sauropia macrorhinus* gen. et sp. nov. Artwork by Caetano Soares.

Although the phylogenetic affinities of *Sauropia macrorhinus* remain a challenging issue due to its putatively immature ontogenetic stage, its small size offers an interesting contribution to our understanding of the composition of Middle Triassic ecosystems in southern Brazil (Fig. 5). Even if *Sauropia macrorhinus* was significantly larger in advanced ontogenetic stages, the record of CAPPA/UFSM 0510 represents the first evidence of an individual with a skull less than 10 mm in length from Middle Triassic deposits in Brazil. The discovery of such a small individual broadens the known fossil record by revealing evidence of distinct ecological niches. Based on its size and tooth morphology, the recovered specimen (CAPPA/UFSM 0510) of *Sauropia macrorhinus* was likely insectivorous or fed on other small invertebrates. Furthermore, it is improbable that large coeval predators, such as *Prestosuchus chiniquensis*^48^, would have targeted such small tetrapods. In contrast, small carnivorous taxa recently described from the same geological unit, such as the approximately 1 m-long pseudosuchian *Parvosuchus aurelioi*^49^, could have preyed upon animals of this size. The expansion of our knowledge of terrestrial food webs from the Middle Triassic is valuable not only because these ecosystems preceded the Carnian Pluvial Episode, but also because they represent the last Mesozoic ecosystems before the dawn of dinosaurs.

## Conclusions

*Sauropia macrorhinus* possesses a unique suite of traits distributed within owenettids and procolophonids. Whereas the traits shared with owenettids are more widely distributed within Procolophonoidea, the traits shared with procolophonids are exclusive of the clade. As a result, *Sauropia macrorhinus* is recovered as a member of Procolophonidae in the phylogenetic analysis. However, the holotype is significantly smaller than any known procolophonid or owenettid specimen, likely representing a skeletally immature individual. Therefore, its ontogenetic status demands caution regarding its phylogenetic interpretations. Regardless of its phylogenetic affinities, this finding expands the known diversity of Middle Triassic parareptiles and offers new insights into their early development. Furthermore, the presence of such a small individual enhances our understanding of the structure and complexity of terrestrial food webs in Middle Triassic ecosystems, prior to the Carnian Pluvial Episode and the rise of dinosaurs.

## Supplementary Information

Below is the link to the electronic supplementary material.

Supplementary Material 1

## Data Availability

The data supporting the findings of this study are included in the Supplementary Information files or are openly available on Figshare at 10.6084/m9.figshare.30940064 (3D model), 10.6084/m9.figshare.30940052 (phylogenetic data matrix), and 10.6084/m9.figshare.30940076 (outputs of the phylogenetic analyses).
