# Supplementary material for: The smallest tetrapod from the Middle Triassic of South America: a new procolophonoid parareptile from the Ladinian of Southern Brazil
Source: Sci Rep. 2026 Jan 28;16:866. doi: 10.1038/s41598-026-35114-3 (PMC12852135; doi:10.1038/s41598-026-35114-3)
Supplement: Supplementary file 1 — Supplementary Material 1 [file 41598_2026_35114_MOESM1_ESM.docx]

**SUPPLEMENTARY INFORMATION**

**The smallest tetrapod from the Middle Triassic of South America: a new procolophonoid parareptile from the Ladinian of Southern Brazil**

Rodrigo T. Müller, Lúcio Roberto-da-Silva, Pedro Lucas Porcela Aurélio & Leonardo Kerber

Corresponding author email: rodrigotmuller@hotmail.com

**Outline of contents:**

1. Results of the phylogenetic analyses using implied weighting

2. Character list

3. Phylogenetic data matrix

4. Comparative cranial measurements of procolophonoid specimens

5. References for supplementary information

**1. Results of the phylogenetic analyses using implied weighting**

**
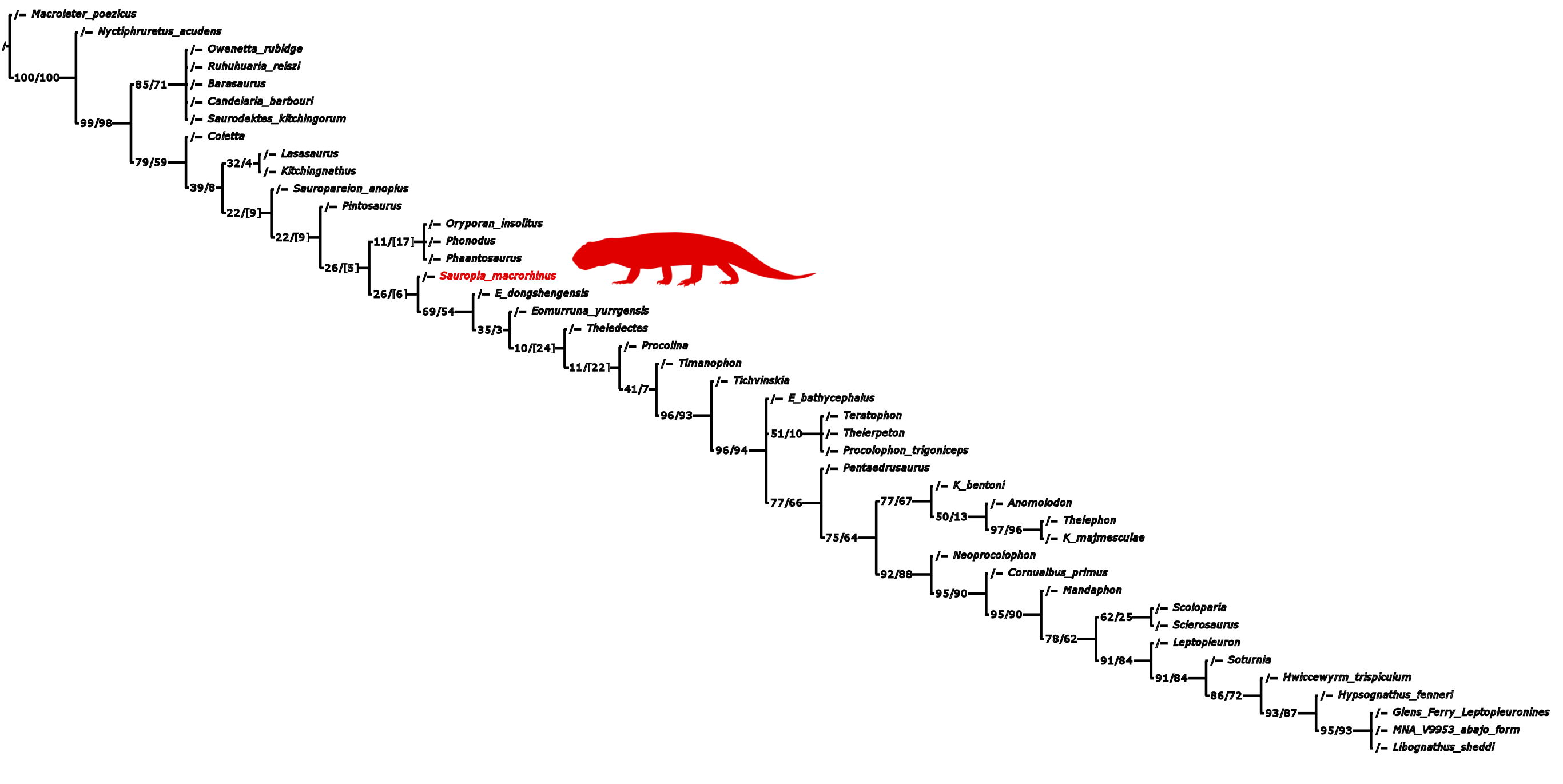
**

**Supplementary figure 1.** Strict consensus tree from the analysis with *k* = 3, depicting the phylogenetic position of *Sauropia macrorhinus* gen. et sp. nov. Values on the branches represent absolute (left) and GC (right) frequencies from symmetric resampling with no-zero weighting.


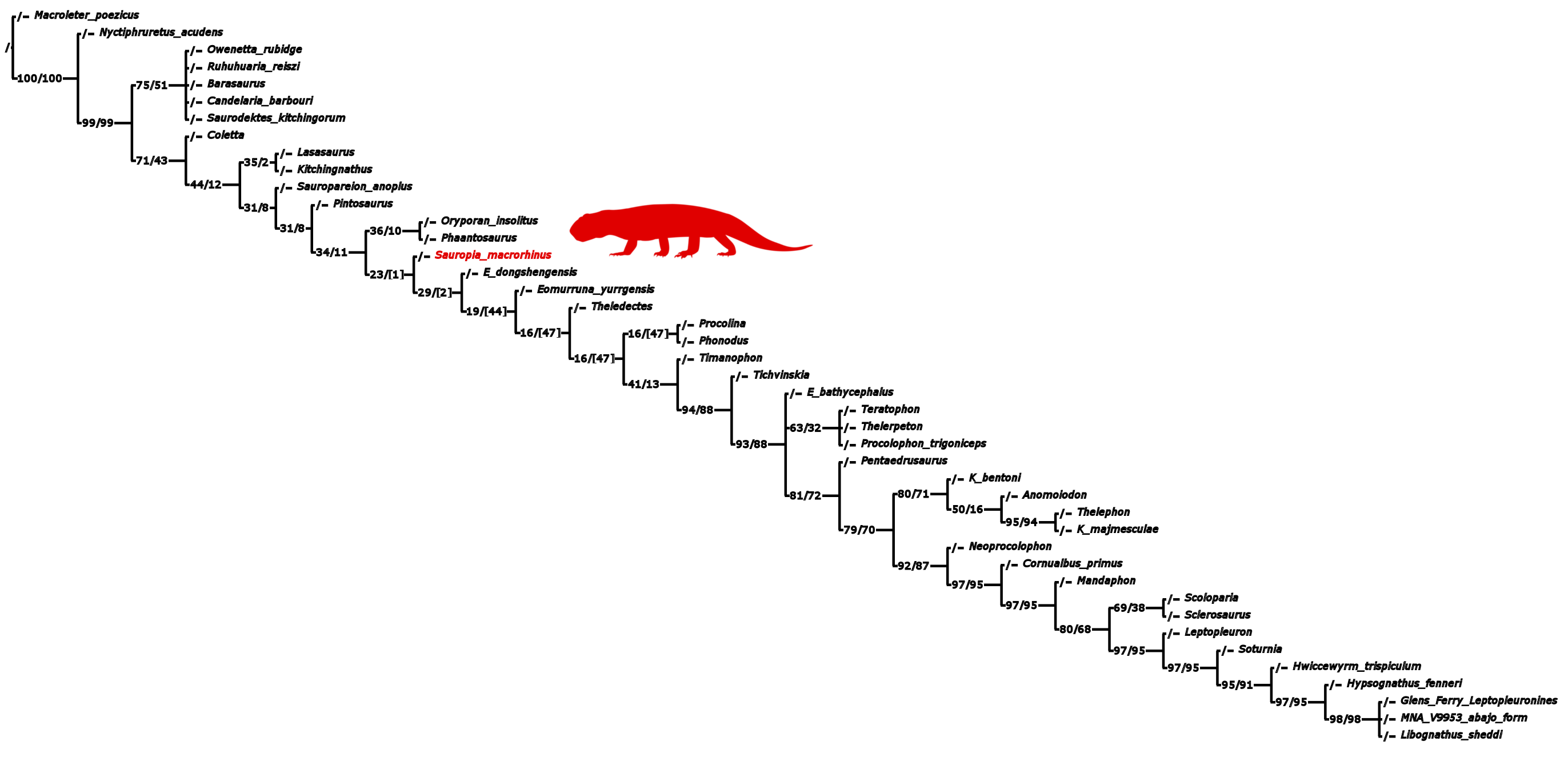


**Supplementary figure 2.** Strict consensus tree from the analysis with *k* = 4, depicting the phylogenetic position of *Sauropia macrorhinus* gen. et sp. nov. Values on the branches represent absolute (left) and GC (right) frequencies from symmetric resampling with no-zero weighting.

**
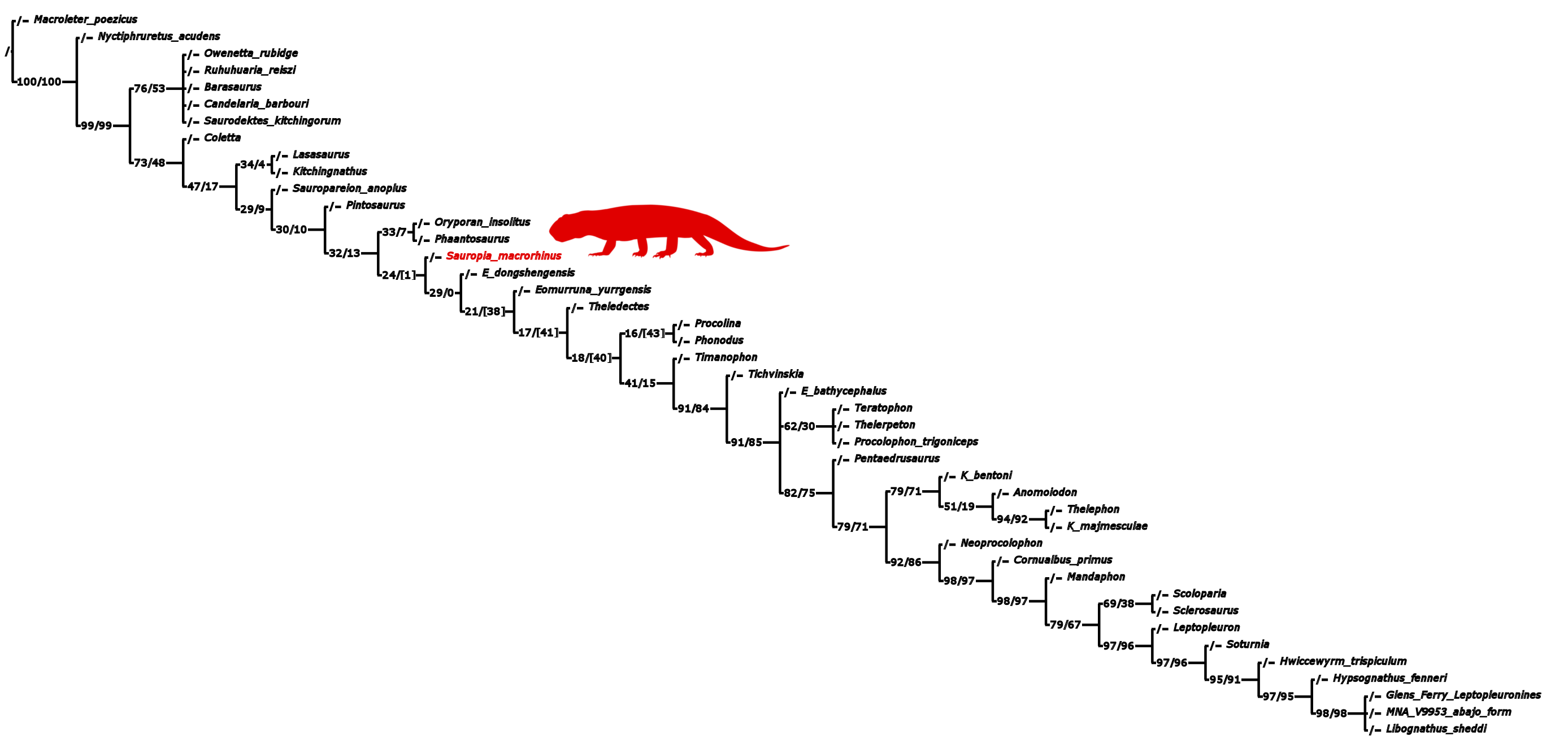
**

**Supplementary figure 3.** Strict consensus tree from the analysis with *k* = 5, depicting the phylogenetic position of *Sauropia macrorhinus* gen. et sp. nov. Values on the branches represent absolute (left) and GC (right) frequencies from symmetric resampling with no-zero weighting.

**
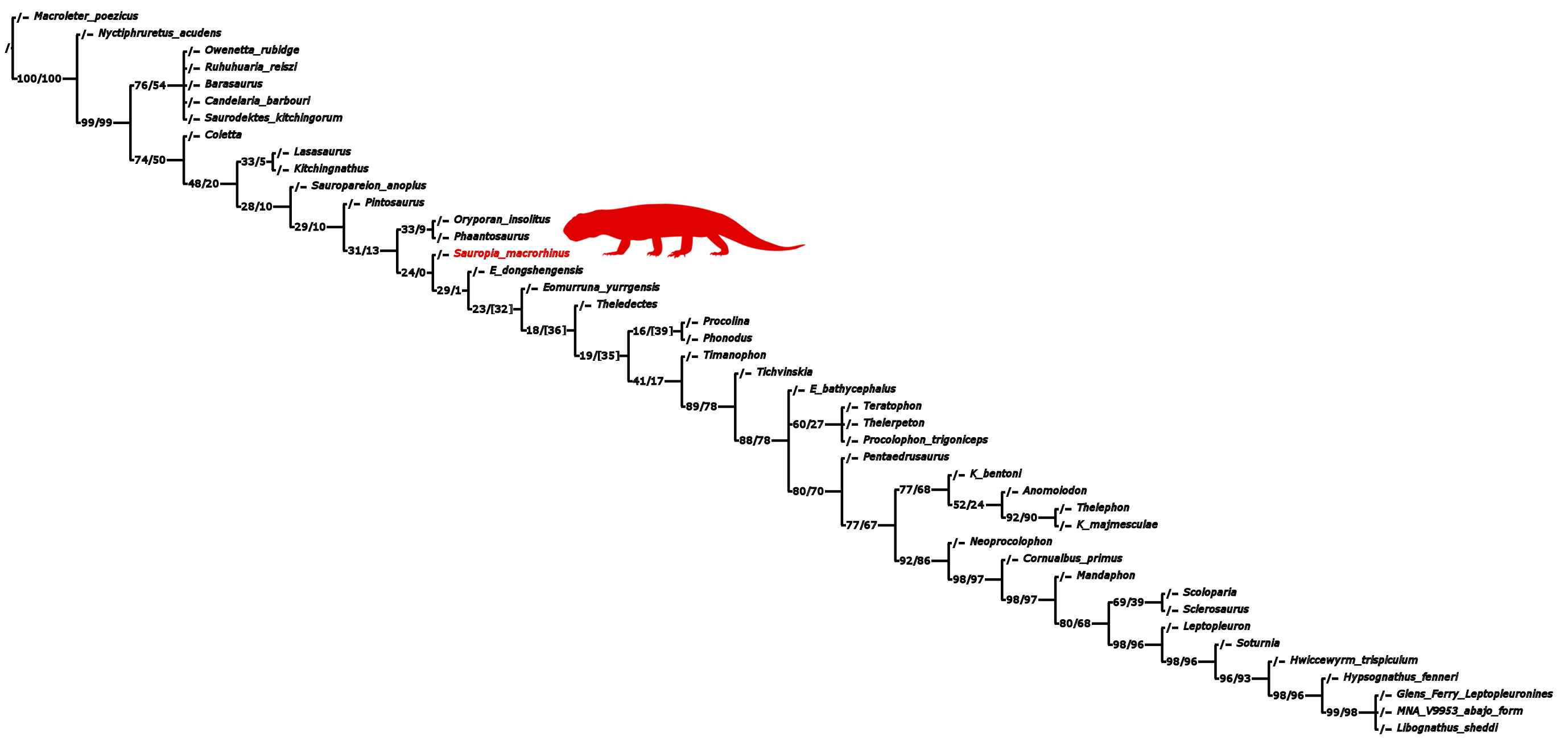
**

**Supplementary figure 4.** Strict consensus tree from the analysis with *k* = 6, depicting the phylogenetic position of *Sauropia macrorhinus* gen. et sp. nov. Values on the branches represent absolute (left) and GC (right) frequencies from symmetric resampling with no-zero weighting.

**
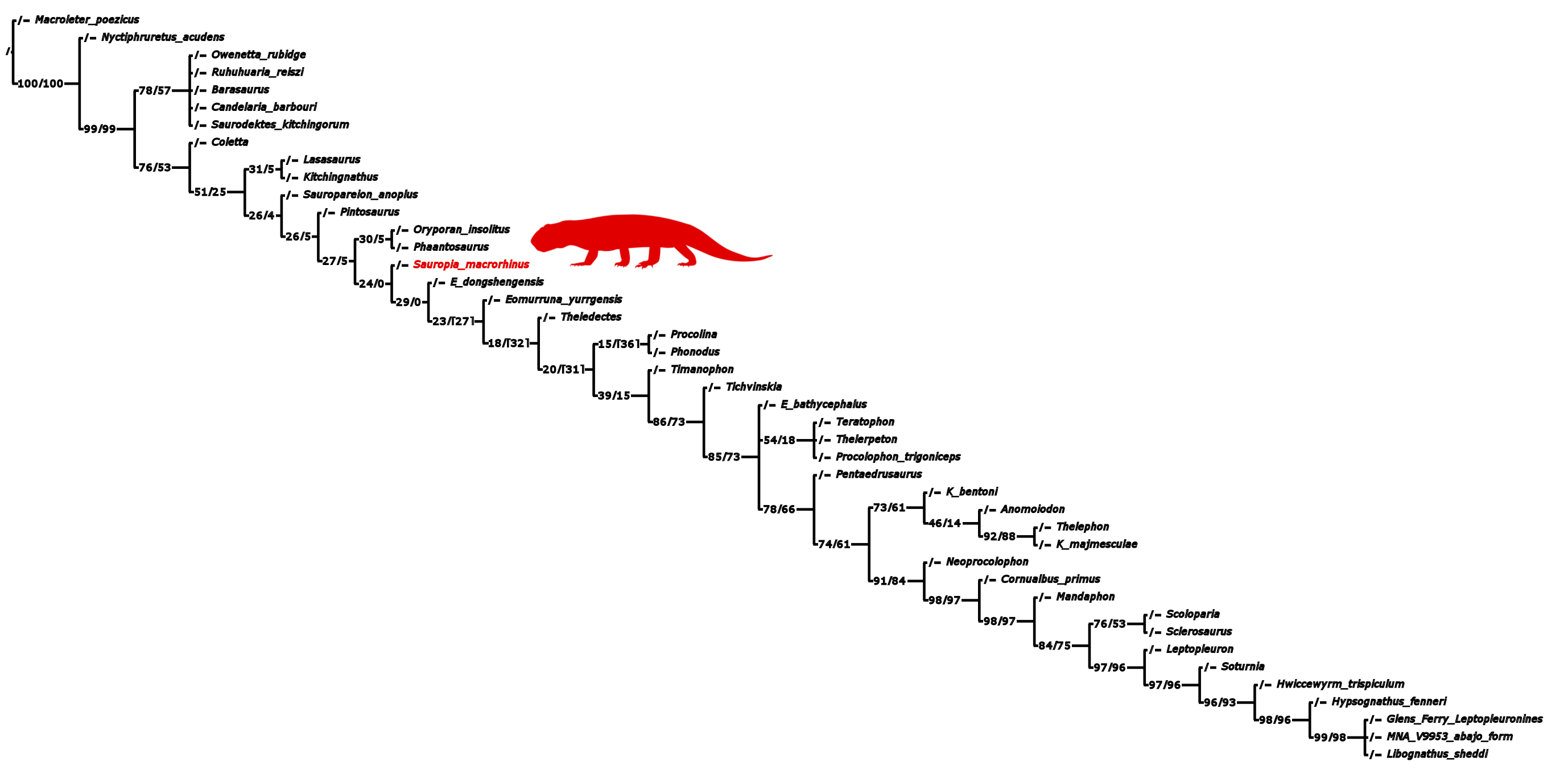
**

**Supplementary figure 5.** Strict consensus tree from the analysis with *k* = 7, depicting the phylogenetic position of *Sauropia macrorhinus* gen. et sp. nov. Values on the branches represent absolute (left) and GC (right) frequencies from symmetric resampling with no-zero weighting.


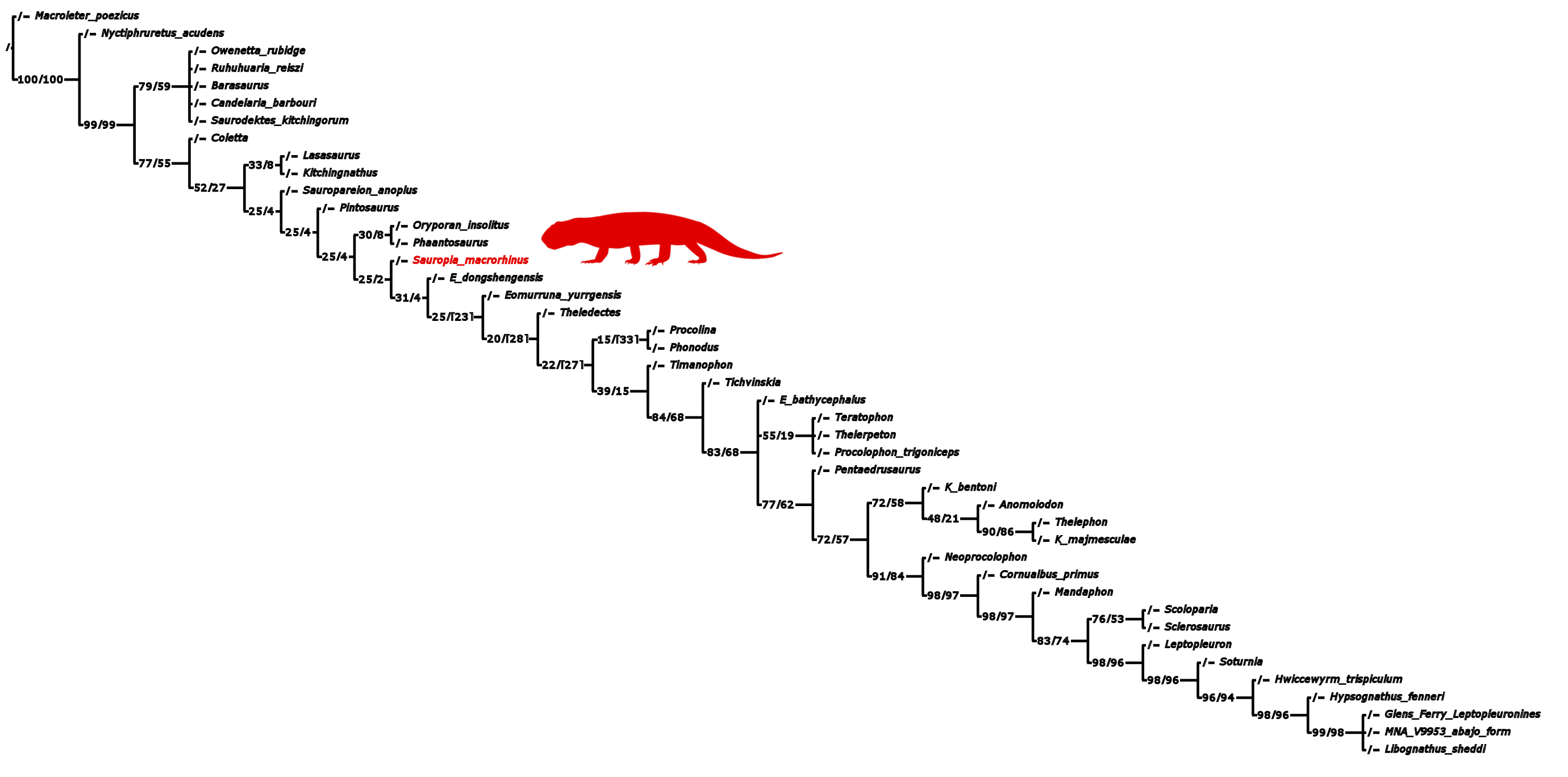


**Supplementary figure 6.** Strict consensus tree from the analysis with *k* = 8, depicting the phylogenetic position of *Sauropia macrorhinus* gen. et sp. nov. Values on the branches represent absolute (left) and GC (right) frequencies from symmetric resampling with no-zero weighting.

**2. Character list**

1. Maxilla, subnarial process:

0) present

1) absent

2. External naris:

0) posterior to first premaxillary tooth

1) anterior to first premaxillary tooth

3. External naris:

0) anteroposteriorly

1) elongated subcircular

4. Wide internarial bar:

0) absent

1) present

5. Snout:

0) long flat

1) deep short

6. Maxilla-nasal, lateral surface, maxillary depression:

0) restricted to anterior narial margin as a narial shelf or absent

1) maxillary depression extends onto lateral surface of anterior snout

7. Prefrontal medial border:

0) straight

1) medial process

2) confined to orbital rim

8. Posterior margin orbitotemporal fenestra:

0) anterior to post margin of pineal foramen

1) posterior-most point of the pineal foramen

2) beyond the posterior border of pineal foramen

9. Pineal opening insertion:

0) shallow fossa

1) flush with dorsal surface

10. Pineal opening shape:

0) rounded

1) teardrop-shaped

2) straight posterior border

11. Postfrontal, if present, bony contacts:

0) contacts fro pa po

1) contacts fro pa pob st

2) contacts fro pa

12. Jugal lateral processes:

0) absent

1) one

2) two

13. Quadratojugal, lateral surface:

0) spineless

1) one spine

2) two spines

3) three spines

14. Squamosal ventral margin:

0) terminates dorsal to quadratojugal

1) at least as far ventrally as quadratojugal

15. Skull roof posterior margin:

0) acute posterior process

1) broad posterior emargination or posterior margin of skull roof flat

16. Postparietal:

0) present

1) absent/fused

17. Supratemporal posterolateral margin:

0) rounded

1) acute

2) prominent spine

18. Vomer width:

0) broader than choana

1) roughly equal or narrower

19. Epipterygoid columella:

0) ends freely

1) contact dorsally prootic and supraoccipital

20. Parasphenoid cultriform process directed:

0) anteriorly and tapers to sharp tip

1) vertically and sharp

21. Basioccipital-quadrate relation:

0) anterior border-quadrate

1) posterior border-quadrate

22. Occipital condyle:

0) basioccipital larger than exoccipital exoccipital

1) forms 1/3 of the condyle

23. Dentary, angle of ventral and dorsal surfaces in posterior jaw:

0) posterior margin of dentary posterior to tooth row nearly parallel

1) posterior margin of dentary posterior to tooth row oblique 20 degrees or more

24. Articular-marginal dentition relation:

0) level to marginal dentition of the dentary

1) articular cotyles ventral to marginal dentition of the dentary

2) articular cotyles far ventral to posteroventral margin of dentary

25. Premaxillary tooth number:

0) five or more

1) four

2) three

3) two

26. Premaxillar teeth:

0) subequal

1) enlarged mesialmost incisor

27. Maxillary teeth with labiolingually expanded bases:

0) absent

1) present

28. Maxilla, dentition, shape or cross section of base of tooth:

0) subcircular or rounded

1) expanded and bulbous

29. Maxillary teeth cervices:

0) not constricted

1) constricted

30. Maxillary teeth cusps:

0) one

1) two

2) monocuspid teeth absent

31. Maxilla, dentition, count in adult specimens:

0) 35–15

1) 12–10

2) 8–6

3) 5

32. Maxillary cheek teeth:

0) not inset

1) inset

33. Maxillary teeth deep occlusal depression or basin:

0) absent

1) present

34. Vomerine dentition:

0) several denticles

1) true teeth

2) entirely absent

35. Vomerine teeth/denticles along posterior medial suture:

0) present

1) absent

36. Palatine dentition:

0) denticles

1) true teeth

2) absent

37. Pterygoid, denticles/teeth:

0) present

1) absent

38. Dentary incisors

0) three or more

1) two

2) one

39. Dentary post-incisiform teeth basal cross section:

0) circular

1) labiolingually expanded

2) mesodistally elongated

40. Dentary post-incisiform teeth cusps number:

0) all teeth monocuspid

1) two present

41. Longitudinal sulcus in pleurocentra:

0) present

1) absent

2) paired sulcci

42. Caudal vertebrae:

0) 20 or more

1) 17 or less

43. Presacral intercentra:

0) present

1) absent

44. Interclavicle longitudinal ridge:

0) smooth

1) prominent

45. Rib cage maximum width

0) short

1) large

46. Interclavicle lateral processes distally:

0) straight

1) posteriorly curved

47. Ectepicondylar foramen or groove on humerus:

0) present

1) absent

48. Entepicondylar process:

0) parallel to shaft

1) perpendicular

49. Entepicondylar foramen on humerus:

0) present

1) absent

50. Non-terminal manual phalanges:

0) long-slender

1) short-robust

51. Ungual-penultimate manual phalanx ratio:

0) equal or shorter

1) longer

52. Illiac anterior surface:

0) convex

1) straight

53. Femur-humerus length ratio:

0) femur longer

1) femur = humerus

54. Femur:

0) slender

1) robust

55. Osteoderms:

0) absent

1) present

56. Lacrimal-ectopterygoid contact:

0) absent

1) present

57. Supraoccipital posterior aspect:

0) dorsally expanded

1) reduced to sagittal pillar

58. Cleithrum:

0) present

1) absent

59. Astragalum and calcaneum:

0) fused

1) not fused

60. Neural spines in thoracic vertebrae:

0) vertical

1) lean posteriorly

61. Supraoccipital-opisthotic occipital contact:

0) cartilaginose

1) ossified

62. Hypoglosal foramina placement:

0) posterior to metotic foramen

1) ventral to metotic foramen

2) anterior to metotic foramen

63. Metotic foramen:

0) surrounded by bone

1) partially surrounded by cartilage

64. Quadratojugal anterior process on ventral embayment:

0) present

1) absent

65. Quadratojugal posterior facial process:

0) absent

1) present

66. Premaxilla, palatal surface, size of foramen prepalatum:

0) absent or small, foramen much smaller than apices of premaxillary teeth

1) large, foramen prepalatum is larger than the apices of the premaxillary teeth

67. Premaxilla, orientation of ascending (supranarial) process in respect to the marginal tooth row of the premaxilla:

0) subvertical at base, then slopes posterodorsally

1) slopes anterodorsally, overhanging tooth row ('rostral process' present)

68. Premaxilla, subnarial process excludes maxilla from margin of external naris:

0) absent

1) present, premaxilla posterodorsally expanded excluding maxilla from narial margin

69. Maxilla, posterior extent of marginal dentition:

0) posterior to or level to anterior orbital margin (or palatal pillar if orbital margin absent)

1) anterior to anterior orbital margin

70. Lacrimal, ventromedial process contributing to the foramen orbitonasale:

0) absent

1) present

71. Maxilla, contribution to orbital margin:

0) absent, maxilla excluded from orbital margin by lacrimal and jugal
1) present, reaches orbital margin in lateral view

72. Jugal, anteroventral process:

0) weak or absent, approximately level or dorsal to maxillary dentition

1) strongly developed, descends further ventrally than the maxillary dentition

73. Jugal, suborbital ramus, length:

0) does not approach the anterior orbital rim

1) approximately level with anterior orbital rim

2) extends far anterior to anterior orbital rim

74. Jugal, free concave ventral margin:

0) absent

1) present

75. Frontals, orbitotemporal crests:

0) absent or weakly developed

1) present

76. Frontals, anteroposterior length at midline relative to parietals:

0) less than 1.5 times parietal length

1) equal or greater than 1.5 times parietal length

77. Postfrontal, ossification:

0) discrete ossification of postfrontal present

1) postfrontal is absent or fused with surrounding bones

78. Quadratojugal, posterior extent:

0) posteriorly level with or anterior to occiput/skull roof

1) extends posterior to skull roof

79. Quadratojugal, ventral extent:

0) does not project strongly further ventrally than level of transverse flange of pterygoid or Meckelian region of the mandible, roughly even to it

1) projects further ventrally than posterior mandible, sometimes sheathing the articular or retroarticular process (or far ventral to transverse flange if mandible not preserved)

80. Quadratojugal, dorsal development covers squamosal otic notch embayment in lateral view:

0) absent

1) present

81. Otic notch, exposure on QJ in lateral view:

0) absent or only present posteriorly

1) present, otic embayment is broadly exposed laterally on QJ

82. Quadratojugal, contact with posteroventral margin of jugal:

0) absent

1) present

83. Quadrate, articular condyles, ventral projection:

0) approximately level to maxillary dentition

1) projects further ventrally than maxillary dentition

84. Palatine, palatine buttress or dorsal platform:

0) absent, dorsal surface of palatine flat or inclined anterodorsally

1) small, dorsal surface of palatine bears dorsal process contributing to less than 75% of the antorbital buttress

2) large, palatine contributes to more than 75% of the buttress forming an antorbital pillar

85. Palatine, posterior margin, position

0) extends posterior to the interpterygoid vacuity

1) approximately level to the interpterygoid vacuity

2) well anterior to the interpterygoid vacuity

86. Dentary, anterior margin, angle between ventral and dorsal surfaces:

0) anterior margin of dentary at symphysis nearly parallel

1) anterior margin of dentary at symphysis oblique by more than 15 degrees

87. Dentary teeth, if two cusps present, distance between cusps:

0) very close, nearly confluent

1) well separated

88. Dentary, incisiform tooth, separated from rest of tooth row by small diastema:

0) absent

1) present

89. Dentary, posteriormost tooth, size:

0) smaller than or equal to other dentary teeth

1) markedly enlarged, largest tooth in mandible

90. Posterior dentary teeth, relationship with lateral edge of jaw:

0) not inset, teeth are almost flush with lateral surface of dentary
1) inset, such as that the tooth row is medial to the lateralmost point of the dentary

**3. Phylogenetic data matrix**

Macroleter_poezicus 000000000000000000??00000100000000000?????0?0??????000000??0???00?00000000000000100010????

Nyctiphruretus_acudens 000000000000000000?0000000000000?00000000000000000000000?00?00000000001000100000100000?000

Coletta 00100100000000?0?0????0?10000000?11??0?0???????????????????????00?000?000101000010000??0?0

Pintosaurus 10???0???????????0????0?20000010?111??00????????????????0?????????000??0?0???????????0???0

Sauropareion_anoplus 1???0101002001000000100010000010?1110000????0011011111?100001??01??0010001100000100000?000

Phaantosaurus 10101100??[12]0?????1?????020110010?101?010?????????????????????????0000?000??10000?10?00?000

E_dongshengensis ?????11????0?????1????????000020?1?1??00????????????????????????????01??0?????????11?0??00

Theledectes ??????1210?00?01??????0?21010020????0100???????00???????????????????0?00011110???1???0?00?

Tichvinskia 1010010210?0010101000000211101210101?011?0??1?1101?011011110010100100100011100000111001001

Timanophon ?????1120120010101?010002?11012001010011??????1?0???????11??010010??0100011100001111001000

K_majmesculae 1111?11201?0?10111????012111012101010011???????????????1???1???1111001?00?10?0000111200011

K_bentoni 1??110?201?011011???1?012?110121021?11[012]1??1??1110????????????????11101001010?1000111200011

Thelephon ??????1201200?011?????0????1012101?10??????????????????????????11???0?0001100000011??0??01

E_bathycephalus 11111102102011??21??10002111012101010011???????????????????????100110?00111100000111?01001

Procolophon_trigoniceps 1111110110201101210010002111012101[01]1001101011111011011011110120100110100111100000111101001

Thelerpeton 1?111?011020110121??1?00211101210101001????1?1???11????????????10??10?0011110?000111?0?0?1

Teratophon 111111011020110121?0100121110121010100?????111110?????01???????10??10100111101100111???0?1

Pentaedrusaurus 11111?0212?0110111????0121110121?11101111?01?111010?110???11???100110?00111000000111??0001

Neoprocolophon ??1111?21[02]?01?11[12]1?????1?????121?11?0??????????????????????????10??????0?11?00?0011???????

Sclerosaurus ???????210?02?1?2???????3?11?[12]21??????1111011?110101111???0?????0???0??0?????00?01???01001

Scoloparia ?????0021??22???2?????1?311[01]12210????111??????????????1?????????011?0?00200?100?011??01001

Leptopleuron 1111000210?120112111?11131111231011202112001111?0???1?011101121?01110100201010010111200001

Soturnia 111100???????????1????1131111231121212201?1?????0??????????1?????10?1?002????????????0?011

Hypsognathus_fenneri 1011002210?230112111111231111131121212?11?1?1???0?????0011?1121?01010101200010110112201111

Phonodus 1?1?111????001???1?????????10??0?1121??????????????????1????????1?01010000???00011011?????

Kitchingnathus 10??0101000000010?????00[12]01001000?????11???????????????????????0??100?0001??00001001?01000

Lasasaurus ???????????000????????00??1101?00???0?????0101?010???????0?????0????0?1001???00?100????0??

Anomoiodon ???1??1211?00?011?????01???1?1[23]1??????1???0???1?0???0?0???01????????0?1?1110??0?0??1?00011

Procolina 11???1???????????1????0?211100[23]0?1?20100??0??????????????????????0?00?????????0????100?001

Mandaphon 11110?02112020012???1?123111?12101???????????1????????0???????????110?0020000001011??0???1

Eomurruna_yurrgensis 1110011210?00001010?100020010020?10101001?01?11101001101101001001010010001110000011100?001

Oryporan_insolitus ?????1??????00??0?????0???110110?101002?????????????????????????????0?????????001????00000

Saurodektes_kitchingorum 00000010001000000000000000000000?000000010000100100100000[01]00100000000010101100?0100000?000

Candelaria_barbouri 0000001000100?0?0???0?0?00000000???????????????????????????????00??00?0010110000100??0????

Barasaurus 000000000010000000?0000000000000?0001000100001001001000?0?00????0000000010110000100000?001

Hwiccewyrm_trispiculum 101100021??12?1121???011311102311????2011??1?1110??0?1?01??11???01010???201??00??1?1?00101

Libognathus_sheddi 1011000?????3????1????1?3111123112121211???????????????0????????01011101200???1?0??2211101

MNA_V9953_abajo_form 1011000210?2301?2???????31111231121???????????????????????????????011??12000?011011???????

Glens_Ferry_Leptopleuronines 101?000????230??21????1231111231121??211???????????????0????????01011101200??0110112211101

Sauropia_macrorhinus 000?1??2??????0?0???????200000?0??????????????????????????????????10?????????00???0???????

Ruhuhuaria_reiszi 0??????????0??????????0?001000000????010????????????????????????????0??0101??????????0?000

Owenetta_rubidge 00000000001000010??000000000000000000??????????????????0???????00?00000010110000100???????

Cornualbus_primus ????01?21030111111?1??103011112101110112??????????1?????????????????0?0010??0000?11??00011

**4. Comparative cranial measurements of procolophonoid specimens**

The following data were used in the one-sample t-test and the linear regression. Some measurements should be interpreted with caution, as they were derived from the scales provided in the figures.

| **Taxon** | **Clade** | **Specimen** | **Reference** | **Total lenght (mm)** | **Orbitotemp. fenest. lenght (mm)** |
| --- | --- | --- | --- | --- | --- |
| *Barasaurus besairiei* | Owenettidae | P1 | Meckert 1995 | 26.1 | 10 |
| *Ruhuhuaria reiszi* | Owenettidae | CAMZM T997 | Tsuji et al. 2013 | 25 | 13.8 |
| *Candelaria barbouri* | Owenettidae | CAPPA/UFSM 0509 | pers. observ. | 38 | 12.4 |
| *Candelaria barbouri* | Owenettidae | CAPPA/UFSM 0255 | pers. observ. | 43 | 15 |
| *Candelaria barbouri* | Owenettidae | UFSM 11131 | Cisneros et al. 2004 | 39 | 12.4 |
| *Candelaria barbouri* | Owenettidae | UFSM 11076 | Cisneros et al. 2004 | 42 | 13.8 |
| *Owenetta rubidge* | Owenettidae | SAM PK K 7582 | Reisz & Scott 2002 | 29 | 12 |
| *Owenetta rubidge* | Owenettidae | RC 50 | Reisz & Scott 2002 | 33.5 | 12.7 |
| *Saurodektes kitchingorum* | Owenettidae | BP/1/4195a | Reisz & Scott 2002 | 28 | 11.5 |
| *Coletta seca* | Procolophonidae | GHG 228 | Modesto et al. 2002 | 29.7 | 12 |
| *Sauropareion anoplus* | Procolophonidae | SAM-PK-11192 | Modesto & Daminai 2007 | 32.9 | 15.7 |
| *Procolophon trigoniceps* | Procolophonidae | CAPPA/UFSM 0189 | pers. observ. | 59.7 | 31.1 |
| *Procolophon trigoniceps* | Procolophonidae | UFSM 11409a | pers. observ. | 90.5 | 35.7 |
| *Procolophon trigoniceps* | Procolophonidae | BM(NH) R. 1949 | Carrol & Lindsay 1985 | 40.1 | 17 |
| *Procolophon trigoniceps* | Procolophonidae | BM(NH) R. 4087 | Carrol & Lindsay 1985 | 42.5 | 16.7 |
| *Procolophon trigoniceps* | Procolophonidae | BM(NH) R. 5483 | Carrol & Lindsay 1985 | 24.7 | 10.9 |
| *Mandaphon nadra* | Procolophonidae | NMT RB167 | Tsuji 2017 | 35 | 17 |
| *Eomurruna yurrgensis* | Procolophonidae | QMF 59501 | Hamley et al. 2020 | 29 | 14.5 |
| *Eomurruna yurrgensis* | Procolophonidae | QMF 49512 | Hamley et al. 2020 | 24.9 | 11.8 |
| *Eomurruna yurrgensis* | Procolophonidae | QMF 49510 | Hamley et al. 2020 | 28.6 | 14.5 |
| *Eomurruna yurrgensis* | Procolophonidae | QMF 49497 | Hamley et al. 2020 | 29.3 | 12.8 |
| *Cornualbus primus* | Procolophonidae | UFSM 11607 | Silva-Neves et al. 2024 | 36 | 19 |
| *Hypsognathus fenneri* | Procolophonidae | YPM 55831 | Sues et al. 2000 | 71.6 | 37.35 |
| *Hypsognathus fenneri* | Procolophonidae | NSM 998GF45.1 | Sues et al. 2000 | 34 | 18.4 |
| *Kapes majmesculae* | Procolophonidae | PIN 4365/40 | Novikov & Sues 2004 | 30.5 | 14.7 |
| *Leptopleuron lacertinum* | Procolophonidae | BMNH R3919 | Säilä 2010 | 47 | 23.6 |
| *Leptopleuron lacertinum* | Procolophonidae | BMNH R4779 | Säilä 2010 | 48 | 24.4 |
| *Leptopleuron lacertinum* | Procolophonidae | ELGNM 1978.718 | Säilä 2010 | 32 | 18.8 |
| *Leptopleuron lacertinum* | Procolophonidae | ELGNM 1920.5 | Säilä 2010 | 39 | 19.5 |
| *Leptopleuron lacertinum* | Procolophonidae | GPIT/AM/00682 | Säilä 2010 | 43 | 23 |
| *Sauropi macrorhinus* | cf. Procolophonidae | CAPPA/UFSM 0510 | pers. observ. | 9.5 | 5.7 |

**5. References for supplementary information**

1. Carroll RL, Lindsay W (1985) Cranial anatomy of the primitive reptile *Procolophon*. *Can. J. Earth Sci.* **22**, 1571–1587.
2. Cisneros JC, Damiani R, Schultz C, da Rosa A, Schwanke C, Neto LW, Aurélio PL (2004) A procolophonoid reptile with temporal fenestration from the Middle Triassic of Brazil. *Proc. R. Soc. Lond. B* **271**, 1541–1546.
3. Hamley T, Cisneros JC, Damiani R (2021) A procolophonid reptile from the Lower Triassic of Australia. *Zool. J. Linn. Soc.* **192**, 554–609.
4. Meckert D (1995) The procolophonid *Barasaurus* and the phylogeny of early amniotes. [Unpublished PhD thesis, University of the Witwatersrand].
5. Modesto SP, Damiani R (2007) The procolophonoid reptile *Sauropareion anoplus* from the lowermost Triassic of South Africa. *J. Vertebr. Paleontol.* **27**, 337–349.
6. Modesto SP, Damiani RJ, Sues HD (2002) A reappraisal of *Coletta seca*, a basal procolophonoid reptile from the Lower Triassic of South Africa. *Palaeontology* **45**, 883–895.
7. Novikov IV, Sues HD (2004) Cranial osteology of *Kapes* (Parareptilia: Procolophonidae) from the Lower Triassic of Orenburg Province, Russia. *Neues Jahrb. Geol. Palaontol. Abh.* **232**, 267–281.
8. Reisz RR, Scott D (2002) *Owenetta kitchingorum*, sp. nov., a small parareptile (Procolophonia: Owenettidae) from the Lower Triassic of South Africa. *J. Vertebr. Paleontol.* **22**, 244–256.
9. Säilä LK (2010) Osteology of *Leptopleuron lacertinum* Owen, a procolophonoid parareptile from the Upper Triassic of Scotland, with remarks on ontogeny, ecology and affinities. *Earth Environ. Sci. Trans. R. Soc. Edinb.* **101**, 1–25.
10. Silva-Neves E, Da-Rosa ÁAS, Modesto SP, Dias-da-Silva S (2024) *Cornualbus primus* gen. et sp. nov.: a new procolophonid (Reptilia: Parareptilia) from Upper Triassic of South America, first tetrapod from the Passo das Tropas Member of the Santa Maria Supersequence. *J. Syst. Palaeontol.* **22**, 2373116.
11. Tsuji LA (2017) *Mandaphon nadra*, gen. et sp. nov., a new procolophonid from the Manda Beds of Tanzania. *J. Vertebr. Paleontol.* **37**, 80–87.
12. Tsuji LA, Sobral G, Müller J (2013) *Ruhuhuaria reiszi*, a new procolophonoid reptile from the Triassic Ruhuhu Basin of Tanzania. *C. R. Palevol* **12**, 487–494.
